# Editorial for the Special Issue on “Nucleic Acid Architectures for Therapeutics, Diagnostics, Devices and Materials”

**DOI:** 10.3390/nano9070951

**Published:** 2019-06-30

**Authors:** Justin R. Halman, Kirill A. Afonin

**Affiliations:** Nanoscale Science Program, Department of Chemistry, University of North Carolina at Charlotte, Charlotte, NC 28223, USA

The use of nucleic acids (RNA and DNA) offers a unique and multifunctional platform for numerous applications including therapeutics, diagnostics, nanodevices, and materials. The ubiquity of nucleic acids and their broad functional involvement in various biological processes place them before other biocompatible materials suitable for controlled bottom-up nanofabrication. Systematic elucidation of RNA and DNA folding, physicochemical properties, and relevant biological activities have fueled an increased involvement of nucleic acid-based nanotechnology in various biomedical challenges. 

This special issue assembles 11 original articles (with seven research manuscripts and four reviews) which nicely outline several advances made in the field of RNA and DNA nanotechnology. Unified by the versatile use of the intriguing biopolymers, these manuscripts explore the various facets of nucleic acid nanostructures such as design, production, and characterization of RNA and DNA nanoassemblies [1,2,3,4], rational design of functional molecular machines [5,6,7,8], immunorecognition of nucleic acid nanoparticles [8], in vivo delivery of therapeutic nucleic acids [9,10], and nucleic acid-based biosensors [11]. We anticipate this special issue to be accessible to a wide audience, as it explores not only the biological aspects of nucleic acid nanodesigns, but also different methodologies of their production, their interactions with other classes of biological molecules, physicochemical characteristics, and possible applications.

Drawing inspiration from naturally-occurring structural and long-range interacting RNA motifs, Chopra et al. describe a novel design that combines the internal ribosome entry site of the hepatitis C virus together with the RNA kissing loop motifs to form hexagonal assemblies which are amenable to functionalization with various aptamers [1]. The aptamers include malachite green, PP7, Spinach, and an aptamer against streptavidin, demonstrating the coordination of proteins and small molecules on RNA scaffolds with structural regularity. The use of RNA in this nanodesign also allowed for co-transcriptional production of functional nanoassemblies. 

O’Hara et al. describe an innovative approach, integrating a split aptamer system into internal portions of hexagonal RNA rings [2]. Using a split-Spinach aptamer, the authors demonstrate that the intensity of the fluorescent signal associated with the formation of the complete aptamer is dependent on the formation of the RNA rings, thus allowing researchers to monitor the assembly of nanostructures. 

In a different approach, Yourston et al. describe the use of nanostructured DNA assemblies for DNA-templated production and organization silver nanoclusters (AgNCs) with unique optical properties [3]. Their work expands the protocols for synthesis of AgNCs on various DNA templates and identifies some structural requirements for altering the fluorescent properties of the AgNCs. 

Design and characterization of nanostructured nucleic acids is of paramount importance in both achieving and modulating their functional goals. The majority of characterization techniques of nucleic acid nanostructures involve the use of electrophoretic mobility shift assays, atomic force microscopy, cryogenic electron microscopy, or dynamic light scattering; each approach has its unique benefits and drawbacks. Oliver et al. propose a new approach to characterizing nucleic acid nanoparticles using small angle X-ray or neutron scattering [4]. Their in-depth review examines the structural and chemical requirements for studying various biomolecules in their natural confirmations. It also highlights the multitude of requirements for solution and sample preparations, and discusses the precise data yielded from these techniques. Finally, it discusses the prospects for enhancing the characterization of nanostructured nucleic acid architectures.

Following design and characterization, advances in the use of DNA and RNA nanostructures in devices and molecular machines is highlighted in this issue. One such device is the DNA nanopore, which offers a unique membrane-bound tool for furthering biophysical research and biosensing. In this issue, Burns and Howorka describe the design and evaluation of a DNA nanopore in various biological milieu [5]. The authors identify parameters for the assembly of a hexameric DNA nanopore using gel electrophoresis and fluorescence spectroscopy, and further confirm its integrity in cell media, as well as its successful integration into lipid membranes.

Beyond the development of biophysical tools, this special issue contains two original manuscripts detailing the use of DNA and RNA nanostructures in the design of molecular logic gates. Using “light-up” malachite green and Broccoli aptamers, Goldsworthy et al. describe the design of robust and precise RNA nanodevices with several Boolean logic functions, including AND, OR, NAND, and NOR capabilities [6]. The binding of the small molecule dyes to their aptamers in the conditional situations causes a fluorescent output based strictly on the input of the designed strands. Using this approach, the authors designed and integrated parallel XOR and AND gates within a single RNA nanoparticle. 

Towards that end, Zakrevsky et al. describe the use of logic gates to mimic complex molecular biological phenomena with the potential for use in cells [7]. In their research, the authors computationally design and characterize DNA/RNA hybrid-based logic gates suited for the conditional release of single- and double-stranded nucleic acids, thus displaying the possibility of therapeutic action. Their set of logic gates offers an eclectic approach inspired by molecular beacons and other trigger-responsive multi-stranded assemblies. 

The concept of smart-responsive or logic-based nucleic acid nanoassemblies is further expanded in the review by Chandler and Afonin [8]. In their review, several advances in the design and function of stimuli-responsive DNA and RNA nanoconstructs are discussed, including simple trigger/activation designs of shape-switching nanoparticles with controlled immunomodulatory properties. Furthermore, they outline the potential of using these dynamic nucleic acid nanoparticles for the conditional stimulation of an immune response, which has been determined to be dependent on the structure and composition of the given nucleic acid nanoparticle.

As therapeutics, nanostructured nucleic acids hold great potential in the treatment of various maladies. Gwak and Lee describe the use of a cationic amphiphilic co-polymer nanoparticles to efficiently deliver plasmid DNA, as well as a small molecule anti-viral therapy for successful treatment of spinal cord injuries using “suicide gene therapy” [9]. The authors further describe the stability of the nanoparticles over time and demonstrate their use in animal models.

In the same regard, Caffery et al. describe in depth the possibility of treating glioblastomas with a combination of gene therapy and various nanoscale carriers [10]. Their comprehensive review discusses both the various therapeutic nucleic acids as well as the various delivery platforms to deliver these therapeutic cargos. The authors also discuss possible limitations to each of these approaches, as well as the challenges of crossing the blood brain barrier.

Finally, this special issue features a review by Sun et al. on the use of genetically encoded RNA-based molecular sensors (GERMS) [11]. The review provides a well-rounded outline of the various design principles and applications for GERMS including their most common use of intracellular imaging. The authors go on to discuss the outlook for this novel technology and potential milestones towards developing next generation RNA based sensors.

Together, this special issue details several important advancements in the field of nucleic acid nanotechnology by focusing on design and characterization, use in devices and molecular machines, and implementation into therapeutics and sensors. The works enclosed here demonstrate the various possible applications for these burgeoning technologies.
